# Optimized Extraction Method for Neutral Cannabinoids Quantification Using UHPLC-HRMS/MS

**DOI:** 10.3390/biom15020246

**Published:** 2025-02-08

**Authors:** João Victor Meirelles, Débora Cristina Diniz Estevam, Vanessa Farelo dos Santos, Henrique Marcelo Gualberto Pereira, Tatiana D. Saint Pierre, Valdir F. Veiga-Junior, Monica Costa Padilha

**Affiliations:** 1 Departamento de Química, Pontifícia Universidade Católica do Rio de Janeiro, Rio de Janeiro 22451-045, RJ, Brazil; jvmeirelles18@gmail.com (J.V.M.); tatispierre@puc-rio.br (T.D.S.P.); 2 Laboratório Brasileiro de Controle de Dopagem (LBCD), Instituto de Química, Universidade Federal do Rio de Janeiro, Rio de Janeiro 21941-909, RJ, Brazil; debora.estevam@gradu.iq.ufrj.br (D.C.D.E.); vanessa.farelo@pos.iq.ufrj.br (V.F.d.S.); henriquemarcelo@iq.ufrj.br (H.M.G.P.); 3 Department of Chemical Engineering, Military Institute of Engineering, Rio de Janeiro 22290-270, RJ, Brazil; valdir.veiga@gmail.com

**Keywords:** *Cannabis* herbal extracts, Full Factorial Design, Box–Behnken design, UHPLC-HRMS/MS, cannabinoids

## Abstract

The *Cannabis* market is experiencing steady global growth. *Cannabis* herbal extracts (CHE) are interesting and sought-after products for many clinical conditions. The medical potential of these formulations is mainly attributed to neutral cannabinoids, such as cannabidiol (CBD), tetrahydrocannabinol (THC), and cannabinol (CBN), and their non-standardized content poses a significant fragility in these pharmaceutical inputs. High-resolution mass spectrometry portrays a powerful alternative to their accurate monitoring; however, further analytical steps need to be critically optimized to keep up with instrumental performance. In this study, Full Factorial and Box–Behnken designs were employed to achieve a multivariate optimization of CBD, THC, and CBN extraction from human and veterinary commercial CHE using a minimum methanol/hexane 9:1 volume and short operational times. A predictive model was also constructed using the Response Surface Methodology and its accuracy was validated. Agitation and sonication times were identified as the most significant operational extraction parameters, followed by the extraction mixture volume. The final setup of the optimized method represented a significantly faster and cheaper protocol than those in the literature. The selected neutral cannabinoids quantification was conducted using ultra high-performance liquid chromatography coupled to high-resolution tandem mass spectrometry (UHPLC-HRMS/MS) with a precision of <15%, accuracy of 69–98%, sensitivity of 23–39 ng kg^−1^, and linearity regarding pharmaceutical requirements. State-of-the-art levels of analytical sensitivity and specificity were achieved in the target quantification due to high-resolution mass spectrometry. The developed method demonstrated reliability and feasibility for routine analytical applications. As a proof-of-concept, it enabled the efficient processing of 16 samples of commercial CHE within a three-hour timeframe, showcasing its practicality and reproducibility, and highlighting its potential for broader adoption in similar scenarios for both human and veterinary medicines.

## 1. Introduction

The historical use of *Cannabis* species (*C. sativa* L. and *C. indica* Lam., Cannabaceae) is extensive, diverse, and controversial. Its commercial potential has been historically known for more than 5000 years, including for paper manufacturing, fiber production, and medicine [1,2]. Despite the historical evidence of its medicinal properties, *Cannabis* suffered a worldwide prohibition campaign in the 1940s, leading to a drastic reduction in its application to research and clinical areas. With the resurgence of scientific and economic interests, medicinal *Cannabis* has been reevaluated and regulated in various countries [2].

The *Cannabis* market is in expressive growth, with continuous projections of expansion and more than 50 different derivative commercially available products [3,4]. In therapeutical scenarios, *Cannabis* herbal extracts (CHEs) are highlighted as the most interesting and sought-after products for a variety of applications in both human and animal healthcare [4,5]. Positive feedback and improvement in health conditions are extensively reported for analgesia, multiple sclerosis, Tourette’s syndrome, epilepsy, Parkinson’s disease, and many others [3,4,5,6,7,8].

The psychoactive and therapeutical potential of *Cannabis* is mainly attributed to neutral cannabinoids, which are bioactive terpene phenolic substances produced in its female inflorescence’s glandular trichomes. Cannabinoid quantification is critical for the quality control and risk assessment of CHEs and other *Cannabis*-based medicines [4,9,10]. Their monitoring using optical and low-resolution detectors presents several limitations that can compromise the accuracy and reliability of the results, such as selectivity to isomers and similar-structure cannabinoids [10,11,12,13]. High-resolution mass spectrometry represents a state-of-the-art analytical alternative that enables sensitive and specific quantification of target substances, as well as an exploratory assessment of these formulations [11,14].

The two main cannabinoids responsible for the pharmacological properties of *Cannabis* and its extracts are tetrahydrocannabinol (THC) and cannabidiol (CBD) [15]. Both exhibit paradoxical effects on the central nervous system, acting synergistically in pharmaceutical formulations: the former is a psychoactive compound with euphoric, antiemetic, and analgesic properties; while the latter is a depressant and antipsychotic with anticonvulsant, anxiolytic, anti-inflammatory, and antioxidant properties [16]. In addition to these, cannabinol (CBN) deserves special attention as a cannabinoid with significant sedative effects and a relevant role in the so-called Entourage Effect—the synergistic interaction of all substances present in *Cannabis* that enhances the therapeutic effects of cannabinoids [17].

In Brazil, ANVISA, the federal agency equivalent to the United States FDA, approved the importation of CBD products in 2015, requiring a prescription from a medical professional. Subsequently, in 2019, the agency established a new category, ‘Cannabis products’, permitting the manufacture and importation of oils and products containing cannabidiol. More recently, the Superior Court of Justice authorized the cultivation of *Cannabis* with low THC content (0.3%) for medicinal purposes [15].

The performance of methods needs to be critically considered for pharmaceutical and bioanalytical requirements [18,19], and understanding the underlying processes is vital. In this context, Design of Experiments (DoE) is a well-established chemometric approach that enables the implementation of the pursued optimum quality in research conditions in a fast, accurate, and economical way [20]. DoE explores the relationships between several explanatory variables and one or more desired responses, enabling actionable insights into the process operation [19,20].

In the present study, a multivariate optimization using DoE was performed to extract three cannabinoids (CBD, THC, and CBN) from CHEs, analyzed using UHPLC-HRMS/MS. The method was validated and applied to 16 commercially available CHEs.

## 2. Materials and Methods

### 2.1. Chemicals and Reagents

For sample preparation, methanol and n-hexane, both gas chromatography grade, were acquired from Tedia (Fairfield, OH, USA). The CBD, THC, and CBN standards were provided by AB Científica Ltd. (Vitória, ES, Brazil), and the carboxy-tetrahydrocannabinol-D_9_ (THC-COOH-D_9_) standard was purchased from Cerilliant Corporation (Round Rock, TX, USA). For UHPLC-HRMS/MS analysis, methanol HPLC grade, formic acid 98–100%, and ammonium formate were provided by Tedia (Fairfield, OH, USA), Merck (Darmstadt, Germany), and Spectrum Chemical (Gardena, CA, USA), respectively. Ultrapure water (18.2 MΩ cm) was obtained from a Millipore Milli-Q purification system (Billerica, MA, USA).

### 2.2. Samples

Four CHEs for veterinary applications were acquired at local markets from Rio de Janeiro, RJ, Brazil. Fourteen herbal oils for human medicine were kindly donated by local *Cannabis* research and costumer-supporting associations based in the state of Rio de Janeiro, RJ, Brazil (Associação Brasileira de Cannabis e Saúde—ACOLHER and Apoio à Pesquisa e Pacientes de Cannabis Medicinal—APEPI). Coconut oil was employed as a sample substitute in the blank solutions, since it is commonly applied as a pharmaceutical vehicle in CHE.

### 2.3. UHPLC-HRMS/MS Instrumentation and Analysis

A Dionex Ultimate 3000 ultra-high-performance liquid chromatography (UHPLC) system coupled to a QExactive Plus hybrid quadrupole Orbitrap mass spectrometer (Thermo Fisher Scientific, Bremen, Germany) equipped with an electrospray ionization (ESI) source was used. Separation was performed in a reversed-phase column (ACE UltraCore 2.5 SuperC18, 50 mm × 2.1 mm; 2.5 µm) at 50 °C, with a constant flow rate of 400 μL min^−1^ and injection volume of 5 µL. A gradient chromatographic run started at 5% of mobile phase B (methanol with 0.1% formic acid) and 95% of mobile phase A (water with 5 mmol L^−1^ ammonium formate and 0.1% formic acid). Mobile phase B was increased to 10% at 0.5 min, 25% at 1 min, and 90% at 6 min. After reaching 100% of B (8 min) and maintaining until 9 min, the initial chromatographic condition was restored from 9.1 to 11.1 min.

The LC effluent was pumped to the mass spectrometer operating in a positive ESI mode, calibrated daily with a manufacturer’s calibration solution (Thermo Fisher Scientific, Bremen, Germany). ESI parameters were defined as a spray voltage of 4.00 kV, S-lens voltage of 80 V, capillary temperature of 250 °C, auxiliary gas heater temperature of 350 °C, nitrogen sheath, auxiliary, and sweep gas set at 30, 10, and 1 arbitrary unit, respectively. Full-scan data were acquired in a range of *m*/*z* 80–800 at a resolution of 70,000 full widths at half maximum (FWHM), automatic gain control (AGC) of 1 × 10^6^, and maximum injection time (IT) of 100 ms. Parallel reaction monitoring (PRM) data were acquired at a resolution of 70,000 FWHM with AGC of 1 × 10^5^, IT of 50 ms, loop count of 2, and isolation window of *m*/*z* 1.0. Data were acquired with a ±5 ppm mass tolerance and processed using Thermo Scientific^TM^ TraceFinder™ 4.1 software (Thermo Fisher Scientific, Bremen, Germany). Precursor ions of *m*/*z* 315.2 (CBD and THC), 311.2 (CBN), and 354.3 (THC-COOH-D9) were fragmented in a higher energy collisional dissociation (HCD) cell with normalized collision energies (NCE) of 40%, 37%, and 35%, respectively.

### 2.4. Optimization of Sample Preparation

#### 2.4.1. Screening Design

A screening of Full Factorial Design (FFD) 2^3^ was performed to select the most critical variables and their levels in the sample preparation method. The sample preparation protocol consisted of an ultrasound-assisted liquid–liquid extraction (UA-LLE) with methanol/hexane 9:1 *v*/*v* as an extractor mixture, as previously successfully reported in the literature [16]. Twenty-five microliters of a CBD, THC, and CBN standard mix at 1.2 mg L^−1^ were added in test tubes and evaporated to dryness in an evaporator at 30 °C under a N_2_ stream. To the dry residue, 100 mg of coconut oil, a blank sample matrix for *Cannabis* extracts, was added and mixed in a mixer (30 s) for complete homogenization.

Methanol/hexane 9:1 *v*/*v* mixture was added to the samples, which were submitted to the UA-LLE in a shaker at 400 rpm, followed by an ultrasound bath. As represented in Supplementary Material Table S1, agitation time in an orbital mixer (5 or 15 min—coded as X_1_), ultrasound time (15 or 45 min—coded as X_2_), and extractor mixture volume (2.50 or 7.50 mL—coded as X_3_) were assessed in two levels as independent variables, with six extra replicates for the central point (10 min, 30 min, 5.00 mL, respectively). When submitting the samples to the ultrasound, it is noteworthy to mention that the bath must be open to avoid heating and, consequently, the degradation of cannabinoids.

After the UA-LLE, the samples were refrigerated at –30 °C for 30 min, centrifugated at 4000 rpm for 20 min, and their organic phases were separated. The final volume was adjusted to 7.50 mL, an aliquot of 975 µL was transferred to a vial, and 25 µL of THC-COOH-D9 10 mg L^−1^ was added as internal standard (IS). UHPLC-HRMS/MS analyzed the samples, and the ratios “Analyte Area/IS Area” were determined for the analytes in each trial. A non-linear regression was used to calculate each variable’s coefficients (b1, b2, and b3) and its relevancy to the experimental protocol.

#### 2.4.2. Modeling Design

A Box–Behnken design (BBD) for 3 variables was performed to find the optimum working region for the sample preparation protocol. As described previously, coconut oil spiked with CBD, THC, and CBN standards was used as a matrix for *Cannabis* extracts. The only difference in sample preparation concerning the screening FFD was the final volume adjustment to 10.0 mL instead of 7.50 mL. To an aliquot of 975 µL of the final solution, 25 µL of THC-COOH-D9 10 mg L^−1^ was added as IS and analyzed using UHPLC-HRMS/MS. Agitation time in shaker (10 or 20 min—coded as X_1_), ultrasound time (5 or 25 min—coded as X_2_), and extractor mixture volume (5 or 10 mL—coded as X_3_) were assessed as independent variables, with five extra replicates at the central point (15 min, 15 min, 7.50 mL, respectively). The ratios “Analyte Area/IS Area” were determined for the analytes in each trial as dependent responses. The BBD experimental design is represented in Supplementary Material.

BBD generalizes a second order predictive model as follows:

(1) $$y=b_{o}+\sum_{i=1}b_{ii}x_{i}^{2}+\sum_{i=1}^{n}\sum_{j=i+1}^{n}b_{ij}x_{i}x_{j};$$

where *y* is the predicted response, *b_n_* are regression coefficients, and *x_n_* are the coded levels of the independent variables [21]. The results of the BBD design were analyzed using regression analysis (Minitab software version 18.1), and Response Surface Methodology (RSM) was constructed in STATISTICA^®^ software version 10.0.0.0. After determining the optimal conditions, the discrepancy between the experimental values and its predicted values was compared to validate the model.

### 2.5. Method Validation

The method was validated according to the Brazilian Health Regulatory Agency (ANVISA) resolution 166/2017 guidelines. Selectivity was evaluated by preparing and analyzing coconut oil, sesame oil, soya oil, and olive oil matrices without fortifying analytes but spiking with the IS.

Linearity was evaluated through the preparation of samples in triplicate at five different concentration levels and scaled using the determination coefficient (R^2^). The data homoscedasticity (Cochran test) and the residual scatter plot were also evaluated. The working range was established from the linearity assessment. Calibration curves were prepared following the established working range in the (i) mixture solvent (methanol: hexane 9:1 *v/v*) for external standard (ES) calibration; (ii) coconut oil matrix for matrix matching (MM) calibration approach; and (iii) using standard addition (SA) in a CHE sample. Angular coefficients were compared for adequate matrix effect investigations using the F-test for variance equality and the *t*-test for analytical inclination equality.

For precision, intra-assay repeatability was estimated by preparing commercial samples spiked with CBD, THC, and CBN in triplicate at three concentration levels (i.e., low, medium, and high), defined from linearity and working range. The results were evaluated through the relative standard deviation (RSD) obtained for each concentration, prepared and injected by a single analyst on a single day and on the same equipment. For inter-assay repeatability, the same sample preparation protocol was conducted by a second analyst on a different day, and the RSD of the combined dataset was calculated.

Accuracy was evaluated through recovery assays for CHE samples’ CBD, THC, and CBN, prepared in triplicate at three concentration levels (i.e., low, medium, and high), defined from linearity and working range. The obtained concentration was compared to the theoretical concentration value. For sensitivity, limits of detection (LOD) and quantification (LOQ) were estimated for the analytes, following Equations (2) and (3), respectively.

(2) $$\mathrm{LOD}=3.3\times \frac{standard\;deviation\;of\;intercept}{analytical\;curve\;slope}$$

(3) $$\mathrm{LOQ}=10\times \frac{standard\;deviation\;of\;intercept}{analytical\;curve\;slope}$$

Three critical parameters on sample preparation were altered by approximately 10% for robustness, and analyte concentrations were monitored for performance comparison. Agitation times of 16 min and 20 min, ultrasound times of 22.5 min and 27.5 min, and solvent volumes of 6.00 mL and 8.00 mL were evaluated.

### 2.6. Analytical Application

The optimized and validated analytical method for CBD, THC, and CBN quantification using UHPLC-HRMS/MS was applied to commercial Cannabis products as a proof-of-concept. A total of 18 CHE samples was analyzed in triplicate.

## 3. Results and Discussion

### 3.1. Chromatographic Separation and Mass Spectrometric Identification

**Figure 1:**
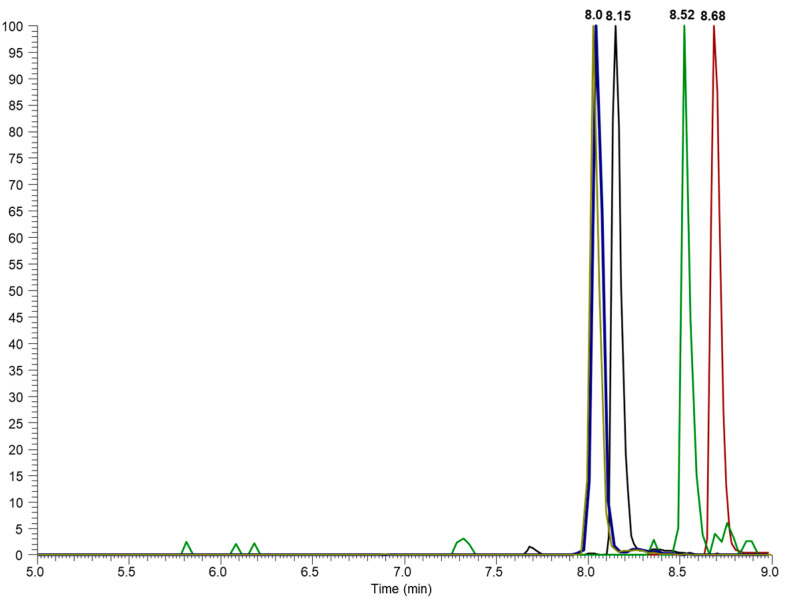
Extracted-ion chromatograms for THC-COOH-D9 (blue), CBD (black), CBN (red), and THC (green) and their retention time.

A baseline separation of all analytes was achieved, as shown in extracted-ion chromatograms (EICs), where specific ions are monitored throughout the run time, filtering the signal according to a predefined *m*/*z* ratio. Unlike the total ion chromatogram (TIC), which displays the sum of all detected ions, the EIC focuses on specific ions, increasing the sensitivity and enabling the identification and quantification of target substances with greater precision (Figure 1). Significant peaks from the substances with a similar structure injected at the same concentration (1 mg/L), and thus exhibiting very similar responses to each other, were detected at retention times (RT) of 8.15, 8.70, 8.52, and 8.03 min for CBD, THC, CBN, and THC-COOH-D9. A comparison of chromatograms obtained for the solvent and the coconut oil showed no variation in RT and RT reproducibility within 0.1% RSD. All these parameters represent the initial characteristics of a fast, selective, precise, and reliable UHPLC method.

Mass spectrometric fragmentation experiments were applied to identify specific fragments for the analytes (Table 1). These findings corroborate data already published in the literature [14,22,23,24], with selective and sensitive mass transitions. CBD and THC are constitutional isomers with similar fragmentation profiles. A neutral loss of 122 Da (*m*/*z* 315 → *m*/*z* 193) can be monitored as a quantifier ion for CBD and THC, which may represent an ether function breaking and a terpene ring cleavage [14,22], while losses of 56 Da (*m*/*z* 315 → *m*/*z* 259) and 180 Da (*m*/*z* 315 → *m*/*z* 135) are monitored as qualifier ions, standing for pentyl groups cleavage and a terpene moiety, commonly found in cannabinoids at a positive ionization mode, respectively [14,22].

In positive mode, CBN (*m*/*z* 311) provides a quantifier product ion at *m*/*z* 223 (80 Da loss), probably given by a dehydrated CBN without a lateral pentyl group [25], and a qualifier product ion at *m*/*z* 241 (70 Da loss) due to aliphatic 5-carbon chain cleavage [25,26]. Another qualifier product ion at *m*/*z* 195 was monitored, a result of a resorcinol moiety and one carbon atom [26] or a sequential pentyl lateral chain and two methyl groups’ losses of a dehydrated CBN [25].

A 46 Da loss (*m*/*z* 354 → *m*/*z* 308), a 152 Da loss (*m*/*z* 354 → *m*/*z* 196), and an 18 Da loss transition (*m*/*z* 354 → *m*/*z* 336) were monitored for THC-COOH-D9. The first one suggests a six-member saturated ring opening [23], while *m*/*z* 196 stands for a terminal C-d_3_ loss from the latter. A typical 18 Da mass loss suggests a common and non-specific H_2_O loss.

### 3.2. Selection of Significant Sample Preparation Parameters

Three operational parameters (agitation time in shaker, sonication time, and extraction mixture volume) from the UA-LLE protocol were investigated. From the experimental batch, non-linear regressions were calculated for each analyte dataset (Table 2).

The agitation time and ultrasound time were the most significant parameters identified in this study. Their isolated contribution and second-order interaction promoted negative effects in all analytes’ normalized signals. Therefore, minimum amounts of both parameters are suggested in an optimized protocol to maximize the analytical signals and minimize the sample preparation cost and time length. LLE methods assisted by agitation and sonication have already been reported with satisfactory results for *Cannabis*-based products in the literature [16,27,28]. Lower polarity solvents (e.g., hexane or dichloromethane) are commonly applied for better oil dissolution followed by a higher solvent extraction, such as ordinary alcohols (e.g., methanol or ethanol), therefore overcoming mass transportation limitations from the matrix [16,28].

The extraction mixture volume was statistically significant for THC and CBN in a third-order interaction with agitation and sonication times, reiterating the importance of modeling all three parameters in this protocol optimization. Despite this statistical significance, there is still a lack of studies investigating the influence of solvent volume on the efficiency of cannabinoid extraction from CHEs. Distinct sample volume ratios ranging from 1:10 to 1:2000 are commonly employed [16,27,28,29,30,31].

### 3.3. Sample Preparation Multivariate Optimization

A three-factor BBD assessed the model for simultaneous CBD, THC, and CBN analytical normalized signals as a function of agitation time, sonication time, and extraction mixture volume. Its coefficients and the model’s figures of merit are summarized in Supplementary Material Table S5.

**Figure 2:**
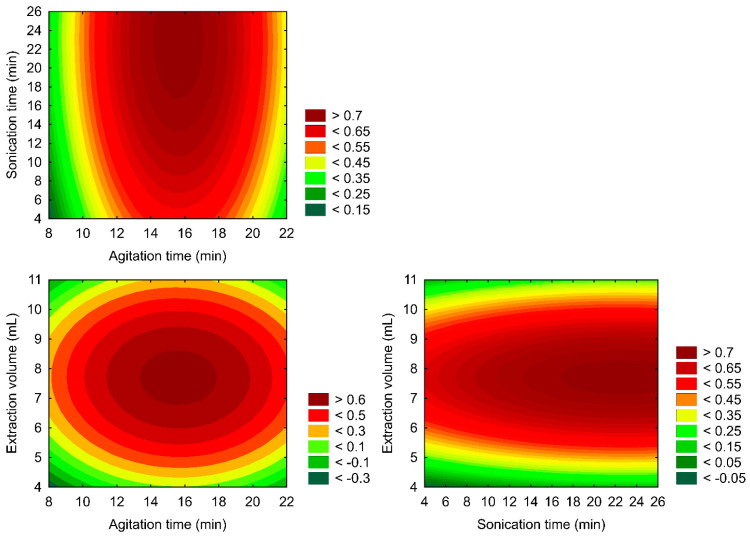
Two-dimensional (2D) Response Surface Methodology for CBD, THC, and CBN normalized analytical signal.

Also, the composed desirability and its two-dimensional (2D) Response Surface Methodology are presented in Figure 2.

The RSM constructed (Figure 2) correlates with the analyte’s composite desirability and variable settings. A composite desirability assesses how well a combination of variables satisfies the goals defined for the responses on a scale of 0 to 1. The optimal region of the sample preparation protocol was localized at 18 min of agitation time, 25 min of ultrasound time, and a 7.00 mL extraction mixture volume (Figure 2), representing a compromise condition with individually optimized rates of 89% (CBD), 80% (THC), and 82% (CBN). These findings represent a faster, cheaper, and simpler protocol compared to previous reports from the literature, with a statistically maximized performance. This optimized setup represents a feasible alternative for routine analysis scenarios, as it could be reproduced in user-friendly processing of a 50-sample batch within three hours.

Most literature reports applied larger extraction mixture volumes and operational times. Carvalho et al. [13] also proposed a UA-LLE sample protocol for CHEs with 10 mL of methanol/hexane 9:1 *v*/*v* and 30 min of sonication time. Yun-Hua Hsu et al. [32] proposed UA-LLE with a larger volume of methanol (8 mL) and a longer sonication time (30 min). Berman et al. [33] established a metabolomic profile from *Cannabis* inflorescences with 10 mL of ethanol as the extraction solvent and 30 min in the ultrasound bath, while Protti et al. [34] managed to extract cannabinoids by applying 10 mL of pure methanol, but 30 min of ultrasound assistance and a second 15 min of agitation were needed. It is noteworthy to mention that Dantas et al. [27] proposed a viable extraction method using 95% ethanol, 5 min of agitation time, and some sonification time. However, no optimization approach was conducted, only a non-standardized trial with distinct conditions. In the present study, the extraction protocol was optimized for CBD, THC, and CBN, and the relationship between operational parameters and analytes response was established. Furthermore, a mathematical predictability model was built.

As an alternative to UA-LEE, Dilute-and-Shoot approaches were also reported with ordinary solvents such as acetonitrile or methanol [13,35,36,37]. However, it is noteworthy to mention that, due to the high matrix complexity, lower detection capabilities are commonly obtained, as are higher interference and co-elution from matrix components [12,38]. Advanced sample preparations, such as UA-LLE and solid-phase extractions, represent reliable and necessary strategies that allow effective detections and sample clean-up [39].

Experimental values from the CBD were compared to the model’s predicted ones to verify the adequacy of the constructed predictive model. The results are summarized in Supplementary Material Table S6. By fitting the mathematical outcome with experimental data, the model’s predictive capacity was validated, and accuracies ranging from 86 to 120% for CBD, 89 to 110% for THC, and 95 to 104% for CBN were observed. These findings corroborate the adequacy of the constructed model for all analytes. The Response Surface Methodology (RSM) built for this sample preparation protocol enhances the efficiency and effectiveness of the experimental processes. A complex system was systematically explored, describing interactions of multiple variables to a targeted outcome. This RSM allows data extrapolations for similar matrices with distinct goals and provides intuitive insights to achieve desired outcomes.

### 3.4. Method Validation

#### 3.4.1. Selectivity and Matrix Effects

There was no evidence of matrix components interfering with the RT or mass transitions of the monitored analytes. At these conditions, no peaks for any mass transitions monitored were detected in blank vegetable oils.

By comparing angular coefficients, significant matrix effects were identified. ES angular coefficients (0.0022 ± 0.0001 for CBD; 0.0023 ± 0.0002 for THC; and 0.0044 ± 0.0003 for CBN) showed statistically significant differences from SA angular coefficients (0.0027 ± 0.0008 for CBD; 0.0028 ± 0.0006 for THC; and 0.0047 ± 0.0008 for CBN), while MM angular coefficients (0.0014 ± 0.0001 for CBD; 0.0014 ± 0.0001 for THC; and 0.0026 ± 0.0002 for CBN) demonstrated statistical similarity with SA angular coefficients.

A complex system, such as natural product extracts, is expected to contain matrix components that can influence the analyte’s detection and, consequently, their quantification. A significant matrix effect was observed in CHEs; therefore, advanced calibration strategies were needed to achieve a reliable analytical approach. Due to its great similarity to the commercial sample’s matrix and powerful compensation of matrix effects, Matrix Matching using coconut oil was proposed and applied. Using this simple and cheap vegetable oil as a matrix simulator, precise (<15% RSD), accurate (recovery 69–98%), and sensitive (LOQ 23–39 ng kg^−1^) analytical responses were achieved with matrix correction.

#### 3.4.2. Linearity and Homoscedasticity

All analytes provided R^2^ > 0.99 in a 25–150 ng g^−1^ concentration range. Cochran’s C-test indicates homoscedasticity (C_critical_ = 0.616; 95% confidence level) for all analytes in all three calibration approaches.

#### 3.4.3. Precision and Accuracy

Satisfactory precision (<15%) and accuracy (70–130%) were obtained for all three analytes in all three concentration levels evaluated, as represented in Table 3, thus reassuring the method’s pertinence to the analytical question.

#### 3.4.4. Sensitivity, Carryover, and Robustness

The LOD (11, 13, and 8 µg kg^−1^) and LOQ (33, 39, and 23 µg kg^−1^) were estimated for CBD, THC, and CBN, respectively. The obtained values were more than 1,000,000-fold lower than the expected concentrations in commercial samples, expressing an adequate quantitative analytical approach. Also, all analytes were not prone to carryover when spiked at a concentration of 300 µg kg^−1^. Analytical blanks were injected after the concentrated sample lacked CBD, THC, and CBN peaks in all monitored mass transitions. The modified conditions evaluated during the robustness assay promoted less than 8% variation in analytes’ normalized intensities. As equivalent analytical performances were observed, the method’s robustness was assured for agitation time, ultrasound time, and solvent volume.

### 3.5. Analytical Application for CHEs

All analyzed sample results are compiled in Table 4. Four CHEs for veterinary medicine (samples 1 to 4) were investigated. The label information of the products disclosed the presence of 6000 µg g^−1^ of CBD and 300 µg g^−1^ of THC. Fourteen human medicine CHEs (samples 5 to 18) were also analyzed.

This batch analysis demonstrates the reliability and user-friendliness of the developed method, providing a feasible alternative for routine analytical scenarios. The developed method enables the efficient processing of more than 50 samples, showcasing practicality and reproducibility.

## 4. Conclusions

The Design of Experiments provided a fast, simple, reliable, and effective approach to optimize the method developed using UHPLC-HRMS/MS. The proposed method identified the agitation time, sonication time, and extraction mixture volume as statistically significant operational parameters for CBD, THC, and CBN extraction, highlighting the interaction between agitation and sonication times.

The full desired response was elucidated in an RSM with proven predictability (86–120%). This analytical challenge was systematically investigated by examining the interactions among multiple variables to achieve a targeted outcome. A 3D surface graph was constructed, fitting operational parameters and analytes responses. The application of the Response Surface Methodology (RSM) enabled data extrapolation to similar matrices with varying objectives and offers intuitive insights for optimizing desired results.

An optimized performance was achieved in a protocol with 18 min of agitation, 25 min of ultrasound, and 7.00 mL of methanol/hexane 9:1 *v*/*v* volume. This protocol is significantly faster and cheaper compared with similar ones reported in the literature.

A Matrix Matching calibration strategy using coconut oils was required due to significant matrix effects in CHEs. The analytical method was successfully validated for pharmaceutical requirements and demonstrated in-agreement precision (<15%), accuracy (69–98%), sensitivity (23–39 ng kg^−1^), and linearity for the analytical problem addressed with matrix correction. The analytical method also demonstrated selectivity and robustness.

The proposed method demonstrated the viability of veterinary and human medicine CHEs analysis. A user-friendly application was conducted as a proof-of-concept that could be feasibly applied to processing a 50-sample batch within three hours.

## Figures and Tables

**Table 1:** CBD, THC, CBN, and THC-COOH-D_9_ molecular formulas, mass transitions, and (N)CE applied to HCD.

Compound	Molecular Formula [M]	Precursor Ion (*m*/*z*) [M + H]^+^	Mass Error (ppm)	(N)CE (%)	Product Ion (*m*/*z*) [M + H]^+^	Mass Error (ppm)
CBD	C_21_H_30_O_2_	315.2	2.85	40	193.1223 ^a^	2.88
					259.1691 ^b^	2.85
					135.1168 ^b^	2.87
THC	C_21_H_30_O_2_	315.2	1.40	40	193.1223 ^a^	1.38
					259.1692 ^b^	1.44
					135.1168 ^b^	1.40
CBN	C_21_H_26_O_2_	311.2	2.73	37	223.1174 ^a^	2.74
					241.1222 ^b^	3.00
					195.1168 ^b^	2.22
THC-COOH-D_9_	C_21_H_19_D_9_O_4_	354.3	1.19	35	308.2570 ^a^	1.52
					196.1411 ^b^	1.99
					336.2520 ^b^	1.18

^a^ = quantifier product ion; ^b^ = qualifier product ion.

**Table 2:** Analysis of Variance (ANOVA) parameters, calculated coefficients, and their *p*-value for screening trials of CBD, THC, and CBN. Significant parameters are highlighted in bold.

	CBD		THC		CBN	
R^2^	0.89835		0.98303		0.98571	
	Coefficient	*p*-Value	Coefficient	*p*-Value	Coefficient	*p*-Value
Curvature	0.015040	0.000000	0.028643	0.000000	0.043007	0.000000
** $b_{o}$ **	0.011825	0.00000318	0.019417	0.00000123	0.028333	0.00000116
** $b_{1}$ **	0.000110	0.818684	**−0.003001**	**0.004565**	**−0.004349**	**0.004472**
** $b_{2}$ **	−0.000957	0.088661	**−0.004502**	**0.000747**	**−0.007873**	**0.000303**
** $b_{3}$ **	0.000397	0.421415	0.000425	0.520152	−0.000223	0.811276
** $b_{1,2}$ **	**0.001423**	**0.025810**	**0.005872**	**0.000214**	**0.009379**	**0.000131**
** $b_{1,3}$ **	−0.000550	0.279443	0.000556	0.407678	0.001043	0.292942
** $b_{2,3}$ **	−0.000055	0.908308	**0.001902**	**0.027101**	0.002009	0.072983
** $b_{1,2,3}$ **	0.001120	0.056671	**0.002298**	**0.013496**	**0.002308**	**0.048208**

**Table 3:** Intra-assay precision (n = 3; two analysts’ datasets), inter-assay precision (n = 6), and recovery for CBD, THC, and CBN. Data are reported as %RSD for precision and % for accuracy.

	CBD			THC			CBN		
Added concentrations (ng g^−1^)	50	100	150	50	100	150	50	100	150
Intra-assay precision (%)	4	4	7	14	6	7	13	14	6
Inter-assay precision (%)	15	10	15	14	7	14	9	15	13
Recovery (%)	86	84	98	69	77	80	76	86	90

**Table 4:** Results for CBD, THC, and CBN concentrations for four veterinary CHEs (1–4) and fourteen human CHEs (5–18). Data are reported as mean ± standard deviation (n = 3).

Sample No.	Description	CBD (mg·g^−1^)	THC (mg·g^−1^)	CBN (µg·g^−1^)
1	CBD:THC 20:1	<LOQ	<LOQ	<LOQ
2	CBD:THC 20:1	0.00004 ± 0.00005	<LOQ	<LOQ
3	CBD:THC 20:1	0.00004 ± 0.000009	<LOQ	<LOQ
4	CBD:THC 20:1	<LOQ	<LOQ	<LOQ
5	THC	0.053 ± 0.003	9.8 ± 0.8	57 ± 11
6	THC	0.048 ± 0.003	14 ± 1	53 ± 6
7	THC	14 ± 4	22 ± 2	195 ± 15
8	THC	21 ± 2	62 ± 6	735 ± 93
9	CBD	14 ± 1	1.52 ± 0.04	40 ± 2
10	CBD	31 ± 5	24 ± 2	96 ± 6
11	CBD	232 ± 23	53 ± 4	276 ± 31
12	CBD:THC 1:1	22 ± 2	20.6 ± 0.6	94 ± 12
13	CBD:THC 1:1	21 ± 1	19 ± 2	51 ± 4
14	CBD:THC 2:1	58 ± 5	19 ± 1	209 ± 3
15	CBD:THC 2:1	52 ± 6	25 ± 2	230 ± 34
16	CBD:THC 2:1	151 ± 16	73 ± 9	589 ± 10
17	Not described	0.060 ± 0.008	1.0 ± 0.1	8 ± 3
18	Not described	0.068 ± 0.007	0.9 ± 0.2	9.4 ± 0.6

## Data Availability

The authors confirm that the data supporting the findings of this study are fully available within the article and its Supplementary Materials. Raw data were generated at LADETEC/UFRJ (Rio de Janeiro, Brazil) and will be available from the corresponding author (M.C.P.) upon request.

## Supplementary Materials

The following supporting information can be downloaded at: https://www.mdpi.com/article/10.3390/biom15020246/s1, Table S1: Screening FFD matrix (2^3^) used for selection of the most important variables in sample preparation. The independent variables (X_1_ to X_3_), and their levels (not coded) are shown in the matrix. Trial 9 is the central point, which was prepared in 6 replicates; Table S2. BBD matrix of experiments (trials) performed to find the robust working region for the sample preparation protocol. The independent variables (from X_1_ to X_3_, not coded) and their levels are shown in the matrix. Trial 13 is the central point, which was prepared in 5 replicates; Table S3. Results from the screening FFD matrix (2^3^) used for the selection of the most important variables in sample preparation. Trial 9 is the central point, which was prepared in 5 replicates; Table S4. Results from BBD experiments (trials) performed to find the robust working region for the sample preparation protocol. Trial 13 is the central point, which was prepared in 5 replicates; Table S5. Analysis of Variance (ANOVA) parameters, p-values of the predictive model, lack-of-fit and calculated not-coded regression coefficients, for CBD, THC and CBN; Table S6. Validation of model predictability for quantification of CBD, THC, and CBN in CHE by UHPLC-HRMS/MS.

## Abbreviations

The following abbreviations are used in this manuscript:

AGC	Automatic gain control
ANOVA	Analysis of Variance
ANVISA	Agência Nacional de Vigilância Sanitária
BBD	Box-Behnken Design
CBD	Cannabidiol
CBN	Cannabinol
CCD	Central Composite design
CHE	*Cannabis* herbal extracts
CID	Collision-induced dissociation
DoE	Design of Experiments
ES	External standard
ESI	Electrospray ionization
FFD	Full Factorial design
FWHM	Full width at half maximum
HCD	Higher-energy Collisional Dissociation
HPLC	High performance liquid chromatography
IT	Injection time
LC	Liquid chromatography
LOD	Limit of Detection
LOQ	Limit of Quantification
MM	Matrix Matching
(N)CE	Normalized collision energy
RSD	Relative standard deviation
SA	Standard addition
THC	Tetrahydrocannabinol
THC-COOH	Carboxy-tetrahydrocannabinol
UA-LLE	Ultrasound assisted liquid-liquid extraction
UHPLC-HRMS/MS	Ultra high-performance liquid chromatography coupled to high-resolution tandem mass spectrometry
USP	United States Pharmacopeia
