# Supplementary material for: Optimized Extraction Method for Neutral Cannabinoids Quantification Using UHPLC-HRMS/MS
Source: Biomolecules. 2025 Feb 8;15(2):246. doi: 10.3390/biom15020246 (PMC11852687; doi:10.3390/biom15020246)
Supplement: Supplementary file 1 [file biomolecules-15-00246-s001.zip › biomolecules-3456916-supplementary.pdf]

Supplementary Materials

Table S1. Screening FFD matrix (2^3) used for selection of the most important variables in sample preparation. The independent variables (X_1 to X_3), and their levels (not coded) are shown in the matrix. Trial 9 is the central point, which was prepared in 6 replicates.

Trial	X_1 - Agitation Time (min)	X_2 – Sonication time (min)	X_3 - Extraction volume (mL)
**1**	5	15	2.50
**2**	15	15	2.50
**3**	5	45	2.50
**4**	15	45	2.50
**5**	5	15	7.50
**6**	15	15	7.50
**7**	5	45	7.50
**8**	15	45	7.50
**9 (n = 6)**	10	30	5.00

Table S2. BBD matrix of experiments (trials) performed to find the robust working region for the sample preparation protocol. The independent variables (from X_1 to X_3, not coded) and their levels are shown in the matrix. Trial 13 is the central point, which was prepared in 5 replicates.

Trial	X_1 - Agitation Time (min)	X_2 - Sonication time (min)	X_3 - Extraction volume (mL)
**1**	10	5	7.50
**2**	20	5	7.50
**3**	10	25	7.50
**4**	20	25	7.50
**5**	10	15	5.00
**6**	20	15	5.00
**7**	10	15	10.0
**8**	20	15	10.0
**9[a]**	15	5	5.00
**10**	15	25	5.00
**11**	15	5	10.0
**12**	15	25	10.0
**13 (n = 5)**	15	15	7.50

Table S3. Results from the screening FFD matrix (2^3) used for the selection of the most important variables in sample preparation. Trial 9 is the central point, which was prepared in 5 replicates.

Trial	CBD (Area/IS)	THC (Area/IS)	CBN (Area/IS)
**1**	0.015	0.042	0.066
**2**	0.016	0.027	0.041
**3**	0.013	0.022	0.032
**4**	0.015	0.022	0.035
**5**	0.019	0.042	0.064
**6**	0.013	0.021	0.034
**7**	0.012	0.021	0.029
**8**	0.017	0.032	0.045
**9 (n = 6)**	0.012 ± 0.001	0.019 ± 0.002	0.028 ± 0.003

Table S4. Results from BBD experiments (trials) performed to find the robust working region for the sample preparation protocol. Trial 13 is the central point, which was prepared in 5 replicates.

**Trial**	**CBD (Area/IS)**	**THC (Area/IS)**	**CBN (Area/IS)**
**1**	0.009	0.012	0.028
**2**	0.007	0.012	0.031
**3**	0.006	0.010	0.032
**4**	0.012	0.016	0.039
**5**	0.007	0.009	0.024
**6**	0.007	0.009	0.021
**7**	0.008	0.011	0.024
**8**	0.005	0.011	0.034
**9**[a]	0.007	0.007	0.019
**10**	0.009	0.014	0.037
**11**	0.009	0.014	0.034
**12**	0.007	0.010	0.026
**13 (n = 5)**	0.010 ± 0.001	0.015 ±0.002	0.039 ± 0.005

Table S5. Analysis of Variance (ANOVA) parameters, p-values of the predictive model, lack-of-fit and calculated not-coded regression coefficients, for CBD, THC and CBN.

	**CBD**	**THC**	**CBN**
**R^2**	0.8554	0.8800	0.8571
**R^2 (Adj.)**	0.6695	0.7257	0.6733
**Model**	0.028	0.016	0.027
**Lack-of-fit**	0.080	0.601	0.901
$\boldsymbol{b_o}$	-0.01910	-0.0471	-0.0936
$\boldsymbol{b_1}$	0.001479	0.00222	0.00383
$\boldsymbol{b_2}$	-0.000291	0.000679	0.00249
$\boldsymbol{b_3}$	0.00529	0.01004	0.02052
$\boldsymbol{b_{1,2}}$	0.000037	0.000029	0.000018
$\boldsymbol{b_{1,3}}$	-0.000055	0.000006	0.000261
$\boldsymbol{b_{2,3}}$	-0.000033	-0.000107	-000261
$\boldsymbol{b_{1,1}}$	-0.000054	-0.000084	-0.000188
$\boldsymbol{b_{2,2}}$,	0.000001	-0.000008	-0.000017
$\boldsymbol{b_{3,3}}$	-0.000266	-0.000546	-0.001316

Table S6. Validation of model predictability for quantification of CBD, THC, and CBN in CHE by UHPLC-HRMS/MS.

Trial	CBD		THC		CBN	
	Exp.	Predicted (% Accuracy)	Exp.	Predicted (% Accuracy)	Exp.	Predicted (% Accuracy)
**1**	0.009	0.01 (111)	0.012	0.012 (100)	0.028	0.028 (100)
**2**	0.007	0.006 (86)	0.012	0.011 (92)	0.031	0.031 (100)
**3**	0.006	0.007 (117)	0.010	0.011 (110)	0.032	0.032 (100)
**4**	0.012	0.011 (92)	0.016	0.015 (94)	0.039	0.038 (97)
**5**	0.007	0.006 (86)	0.009	0.008 (89)	0.024	0.025 (104)
**6**	0.007	0.008 (114)	0.009	0.010 (111)	0.021	0.023 (110)
**7**	0.008	0.007 (88)	0.011	0.010 (91)	0.024	0.023 (96)
**8**	0.005	0.006 (120)	0.011	0.011 (100)	0.034	0.033 (97)
**9**[a]	0.007	0.007 (100)	0.007	0.007 (100)	0.019	0.018 (95)
**10**	0.009	0.009 (100)	0.014	0.013 (93)	0.037	0.036 (97)
**11**	0.009	0.009 (100)	0.014	0.014 (100)	0.034	0.035 (103)
**12**	0.007	0.008 (114)	0.010	0.010 (100)	0.026	0.027 (104)
**13**	0.010	0.010 (100)	0.015	0.015 (100)	0.039	0.039 (100)
